# Erratum for Uddén et al., “A Nonfunctional Opsonic Antibody Response Frequently Occurs after Pneumococcal Pneumonia and Is Associated with Invasive Disease”

**DOI:** 10.1128/mSphere.00248-20

**Published:** 2020-04-08

**Authors:** Fabian Uddén, Jonas Ahl, Nils Littorin, Kristoffer Strålin, Simon Athlin, Kristian Riesbeck

**Affiliations:** a Clinical Microbiology, Department of Translational Medicine, Faculty of Medicine, Lund University, Malmö, Sweden; b Infectious Diseases, Department of Translational Medicine, Faculty of Medicine, Lund University, Malmö, Sweden; c Unit of Infectious Diseases, Department of Medicine, Huddinge, Karolinska Institutet, Stockholm, Sweden; d Department of Infectious Diseases, Faculty of Medicine and Health, Örebro University, Örebro, Sweden

## ERRATUM

Volume 5, no. 1, e00925-19, 2020, https://doi.org/10.1128/mSphere.00925-19. Table S1: In the original version of this table, the third column, containing data on gender about all studied individuals, was incorrectly ordered. A corrected version of Table S1 has been uploaded to the original article record.

